# Isotropic 3D Nuclear Morphometry of Normal, Fibrocystic and Malignant Breast Epithelial Cells Reveals New Structural Alterations

**DOI:** 10.1371/journal.pone.0029230

**Published:** 2012-01-05

**Authors:** Vivek Nandakumar, Laimonas Kelbauskas, Kathryn F. Hernandez, Kelly M. Lintecum, Patti Senechal, Kimberly J. Bussey, Paul C. W. Davies, Roger H. Johnson, Deirdre R. Meldrum

**Affiliations:** 1 School of Electrical, Computer and Energy Engineering, Arizona State University, Tempe, Arizona, United States of America; 2 Center for Biosignatures Discovery Automation, Biodesign Institute, Tempe, Arizona, United States of America; 3 School of Life Sciences, Arizona State University, Tempe, Arizona, United States of America; 4 Clinical Translational Research Division, Translational Genomics Research Institute, Phoenix, Arizona, United States of America; 5 Department of Physics, Arizona State University, Tempe, Arizona, United States of America; City of Hope National Medical Center and Beckman Research Institute, United States of America

## Abstract

**Background:**

Grading schemes for breast cancer diagnosis are predominantly based on pathologists' qualitative assessment of altered nuclear structure from 2D brightfield microscopy images. However, cells are three-dimensional (3D) objects with features that are inherently 3D and thus poorly characterized in 2D. Our goal is to quantitatively characterize nuclear structure in 3D, assess its variation with malignancy, and investigate whether such variation correlates with standard nuclear grading criteria.

**Methodology:**

We applied micro-optical computed tomographic imaging and automated 3D nuclear morphometry to quantify and compare morphological variations between human cell lines derived from normal, benign fibrocystic or malignant breast epithelium. To reproduce the appearance and contrast in clinical cytopathology images, we stained cells with hematoxylin and eosin and obtained 3D images of 150 individual stained cells of each cell type at sub-micron, isotropic resolution. Applying volumetric image analyses, we computed 42 3D morphological and textural descriptors of cellular and nuclear structure.

**Principal Findings:**

We observed four distinct nuclear shape categories, the predominant being a mushroom cap shape. Cell and nuclear volumes increased from normal to fibrocystic to metastatic type, but there was little difference in the volume ratio of nucleus to cytoplasm (N/C ratio) between the lines. Abnormal cell nuclei had more nucleoli, markedly higher density and clumpier chromatin organization compared to normal. Nuclei of non-tumorigenic, fibrocystic cells exhibited larger textural variations than metastatic cell nuclei. At p<0.0025 by ANOVA and Kruskal-Wallis tests, 90% of our computed descriptors statistically differentiated control from abnormal cell populations, but only 69% of these features statistically differentiated the fibrocystic from the metastatic cell populations.

**Conclusions:**

Our results provide a new perspective on nuclear structure variations associated with malignancy and point to the value of automated quantitative 3D nuclear morphometry as an objective tool to enable development of sensitive and specific nuclear grade classification in breast cancer diagnosis.

## Introduction

Breast cancer is a highly heterogeneous disease characterized by several clinical and molecular variations [1]–[3]. It presents a major health concern worldwide and remains the most common cancer among women [4] despite decades of extensive research. In the United States alone, about 232,000 newly diagnosed cases and 39,500 deaths are estimated for the year 2011 [5]. Accurate diagnosis of suspicious masses is critical to early detection and management of breast cancer.

Histopathological assessment of nuclear structure by brightfield microscopy following the staining of tissue sections with hematoxylin and eosin (H&E staining) remains the definitive clinical diagnostic approach to determine malignancy. Image contrast arises, in part, due to hematoxylin binding to acidic functional groups in the cell, causing preferential absorption by chromatin and the nuclear envelope. Along with tissue architecture, pathologists qualitatively assess features such as nuclear size, shape, nucleus-to-cytoplasm ratio, and chromatin texture. Factors such as focal plane selection, sample orientation, and the bisectioning of cells during sample preparation may bias the outcome of the diagnosis due to obscuration or incomplete feature detail.

Computerized 2D image analysis enables quantification of nuclear morphology from digital microscopy images. Computerized nuclear morphometry and its relevance as a biomarker for breast cancer detection and progression have been evaluated in a number of studies [6]–[18], but limitations inherent to 2D analyses of histological specimens often produced equivocal mappings between cancer grade and its associated morphometrics. Intuitively, it would seem that cell classification accuracy and, thus, clinical diagnostic power would be increased by analyzing 3D instead of 2D imagery. 3D cell imaging modalities such as confocal microscopy have been applied for nuclear morphometry [19]–[21]. However, the major drawback of such techniques is the generation of pseudo-3D images by stacking parallel 2D image slices (z stacks). With pseudo-3D imagery, computational precision is compromised by technical limitations inherent to the imaging technology, including inferior spatial resolution in the z-axis. Consequently, the accuracy of measurements becomes orientation dependent. Accurate quantitative characterization of nuclear structure by applying 3D analyses of high contrast, high resolution 3D imagery with isometric resolution would facilitate better assessment of morphological changes associated with malignancy. The Cell-CT® imaging platform is based on absorption-mode micro-optical projection computed tomography [22], uses a 24-bit color camera, and enables 3D imaging of biological cells with an isometric resolution of 350 nm. Its merit for precise and sensitive cytometry has been demonstrated previously [23].

We used the Cell-CT® platform (VisionGate, Inc., Phoenix, AZ) followed by automated 3D image analysis to investigate the variations in 3D nuclear structure and coarse chromatin architecture in human breast cancer using three well-characterized cell lines derived from normal, fibrocystic or metastatic carcinoma human breast epithelium. We computed forty-two 3D metrics that describe the morphology and texture of the nuclei, and determined the discriminatory power of features to distinguish among cell types. Nuclear shape analysis revealed four shape categories present in all three cell types, and statistical analysis of nuclear morphometrics revealed several statistically significant variations between the normal and abnormal cells that may provide new perspectives for diagnosis. The inherent intra- and inter-population heterogeneity among cells and cell types is reflected in our results. This study is the first comparative quantitative analysis of 3D nuclear architecture in a mammary epithelial cell model.

## Materials and Methods

### Cell culture

The normal human mammary epithelial cell line HME1-hTERT (referred to as HME1, herein) was procured from American Type Culture Collection (ATCC, Manassas, VA). It was originally derived by reduction mastectomy from a patient without evidence of cancer [24]. The non-tumorigenic MCF-10A and metastatic MDA-MB-231 cell lines were provided by Dr. Thea Tlsty at the University of California, San Francisco, USA. MCF-10A cells were derived from a patient with extensive fibrocystic disease [25]. The MDA-MB-231 cell line was derived from pleural effusion of a patient with metastatic adenocarcinoma [26]. HME1 and MCF-10A cell lines are near diploid but the MDA-MB-231 line is near triploid. Genomic profiles of the abnormal cell lines include a homozygously deleted p16 locus and wild-type p53 in MCF-10A, and deleted p16 and mutant p53 in MDA-MB-231 [27].

We cultured HME1, MCF-10A, and MDA-MB-231 cells according to supplier protocols. Cells were cultured in T75 tissue culture flasks (Corning, Corning, NY) to approximately 80% confluence, at which time they were trypsinized, centrifuged at 113 g for 3 minutes, and resuspended in 2 mL of appropriate medium. The cell viability was determined to be at least 95% using the Countess® cell counter (Invitrogen, Carlsbad, CA).

Henceforth, we refer to the HME1 cell line as ‘normal’ and to the MCF-10A and MDA-MB-231 cell lines as ‘abnormal’.

### 3D imaging using Cell-CT

A detailed description of this procedure is available elsewhere [28]. Briefly, we fixed the cells for one hour at room temperature with CytoLyt (Cytyc, Marlborough, MA) and smeared them onto a clean microscope glass slide as a preparatory substrate (VWR, West Chester, PA) coated with 0.01% Poly-L-Lysine (PLL; Sigma Aldrich, St. Louis, MO). Hematoxylin and eosin solutions were prepared with filtered tap water. We stained the cells for 1–2 minutes (cell line dependent) in aqueous 6.25% weight/weight (w/w) Gill's hematoxylin (Electron Microscopy Sciences, Hatfield, PA) solution, then counter-stained in bluing reagent (Fisher Scientific, Fair Lawn, NJ) for 30 seconds after washing three times with filtered tap water. After three additional washes with filtered tap water, we stained the cells with 1% w/w eosin (Electron Microscopy Sciences, Hatfield, PA). We then dehydrated the cells through an ethanol series (50%, 95%, and 100%) and two washes with 100% xylene. Lastly, we embedded the stained cells into a carrier gel (SmartGel, Nye Lubricants, Fairhaven, MA) and loaded the resulting cell-gel suspension scraped from the glass slide into a 100-µL glass syringe (Hamilton, Reno, NV). To ensure that images reflect cell structure and not staining artifact, it is critical that cells are optimally stained.

We imaged cells serially with the Cell-CT® instrument by flowing the cells suspended in carrier gel through a rotating glass capillary housed in a specialized imaging cartridge. For each cell, we generated a volumetric image by acquiring 500 projection images taken at angular intervals of 0.72 degrees around the cell and subjecting these projection images to mathematical reconstruction algorithms [29]. Prior to reconstruction, we de-noised and aligned the projection images to remove pattern noise artifacts and to compensate for mechanical displacement and run-out of the capillary. We imaged 150 interphase cells from each cell line and used the generated volumetric images for 3D feature extraction and morphometric analyses.

### 3D nuclear morphometry

For 3D morphometry, we quantified cell and nuclear structure with automated 3D image processing algorithms applied to reconstructed monochrome cell images [28]. We de-noised the image, segmented the volumes of interest (VOIs) including the cell, the nucleus, nuclear compartments and the nuclear DNA, and computed a total of forty-two 3D morphological and textural descriptors (features) from the segmented VOIs (see Table 1). To ensure accurate feature computation, a trained operator visually cross-validated the automatically segmented VOIs using VolView software (version 3.2, Kitware, Clifton Park, NY). Computation of these features was adapted from Doudkine et al. [30], wherein the feature definitions are provided.

**Table 1:** List of features.

Feature type (number)		Features
Morphological (9)		cell volume, nuclear volume, nucleus to cytoplasm volume ratio, number of nucleoli, total nucleolar volume, mean nucleolar volume, mean nucleolar margination (from nucleus center), variance in nucleolar margination, nuclear sphericity
Texture (33)	Descriptive texture (5)	integrated optical density, mean optical density, variance in optical density, skew in optical density, kurtosis in optical density
	Discrete texture (24)	number of objects in low, medium and high condensation states (3), volume fraction of each condensation state (3), content (optical density) fraction of each condensation state (3), smoothness of transition between condensation states (3), compactness of each condensation state (4), average distance of voxels in each condensation state from nucleus center (4), average distance of center of each condensation state from the nucleus center (4)
	Markovian texture (4)	energy, contrast, correlation, homogeneity

Textural descriptors provide a means to characterize density variations within the nuclei. We computed descriptive, discrete and Markovian texture descriptors (Table 1). Descriptive texture features statistically characterize the nuclear content based on the nuclear voxel density histogram. Discrete texture features reflect differences within and between nuclear regions of specific optical density range. To compute these features, we divided the nuclear volume into three distinct regions representing low, medium and high optical density. The thresholds separating low- and high-density regions from medium-density regions were set at a single standard deviation around the mean nuclear density. To quantify the distribution (margination) of connected coarse chromatin regions within the nucleus we determined the center (location in 3D space) of low-, medium-, and high-density chromatin clumps by computing their density-weighted center of gravity. Nucleoli are included in the high density region. Markovian features characterize the directional organization of nuclear content in 3D space and can be used to investigate optical density variations at different granularities within the nuclear volume. We used the co-occurrence matrix-based method developed by Haralick, et al. [31] to compute the Markovian features. We examined nuclear density variations over all twenty-six possible orientations (corresponding to the 26-voxel neighborhood) in discrete 3D space at three length scales: 0.44, 0.74, and 1.5 microns. The 0.74-micron length scale corresponded to the smallest average nucleolar width (occurred in MDA-MB-231 nuclei). 1.5 microns was the largest average nucleolar width (occurred in HME1 nuclei). The 0.44 micron length scale was chosen to quantify textural variations at the finest possible length scale.

We used VolView software to produce 3D renderings of the reconstructed cell imagery. These renderings, shown in the figures and supplemental movies, are useful when visually checking the segmentation results, and for providing an interactive means for data exploration and shape or texture evaluation. All segmentation and computations were performed on the reconstructed grayscale data. We performed 3D shape classification on the segmented nuclei to investigate shape heterogeneity within a cell type and variations among the cell types.

### Statistical analysis

We used Origin software (version 8.0, OriginLab, Northampton, MA) to produce histograms that convey the differences in morphometric feature distributions among the three cell types. For ease of visualization, we connected the centers of each histogram bin with cubic splines. We used Origin and Matlab (version 2010a, Mathworks, Natick, MA) for our statistical analyses. We applied the Shapiro-Wilk test to test the normality of the feature data. Based on the outcome of the normality test, we appropriately applied ANOVA (for normally distributed feature data) and Kruskal-Wallis tests (for non-normal feature distributions) to investigate the ability of our computed 3D features to distinguish among the three cell populations. We computed p-values and tested for significance after correcting for multiple comparisons using Scheffe's method.

## Results

### Mammary epithelial cell nuclei exhibited four distinct 3D nuclear morphologies

**Figure 1:**
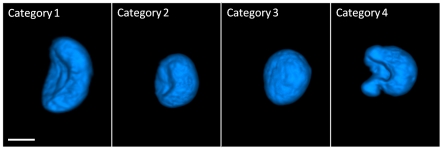
Surface shaded renderings illustrating four nuclear shape categories of human mammary epithelial cells (scale bar = 5 microns).

**Figure 2:**
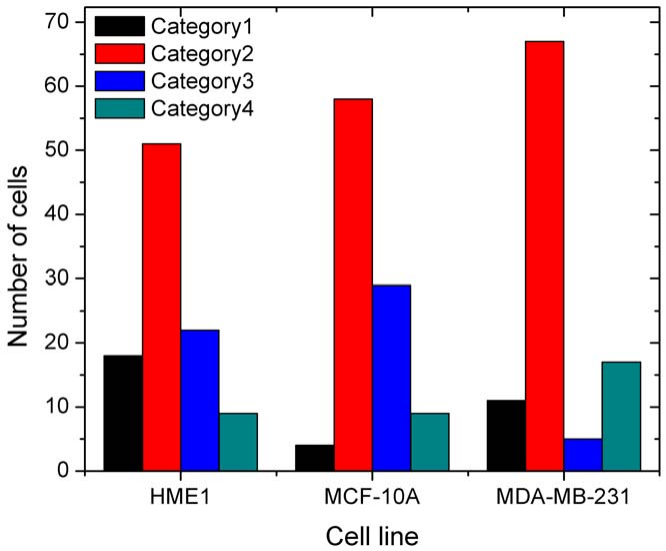
Shape classification of nuclei.

The nuclei from all three cell types could be classified into four distinct nuclear shape categories. These are illustrated as surface-shaded 3D renderings in Figure 1 (see Figure S1 for more detailed illustrations). In contrast to results of prior studies on these cell lines based on 2D or pseudo-3D imagery, we observed four distinct nuclear shape categories. Nuclei in category 1 had a marked concavity and were slender. Nuclei in category 2 had a slight concavity and were bulky. We call shape categories 1 and 2 the mushroom cap morphology. Category 3 nuclei were overall convex. Category 4 included nuclei that had an irregular, distorted shape. All 450 segmented nuclei from the three cell lines were categorized according to the above four shape categories as shown in Figure 2. The nuclear shape classification results in Figure 2 indicated that category 2 (fat mushroom cap with slight concavity and large minor axis) was the most common nuclear shape in all three cell lines. MDA-MB-231 nuclei exhibited the largest fractions of category 4 (irregular) and category 2, and the smallest fraction of convex shape (category 3). Shape heterogeneity within category 4 was greatest in the MDA-MB-231 cell line. MCF-10A nuclei showed the largest fraction of category 3 and the fewest of category 1. HME1 nuclei showed the largest overall shape heterogeneity with respect to the four shape categories (least variation in percentages between the four categories).

### Cell-CT imaging enabled precise structural characterization of micronuclei and multilobular nuclei in metastatic adenocarcinoma cells

**Figure 3:**
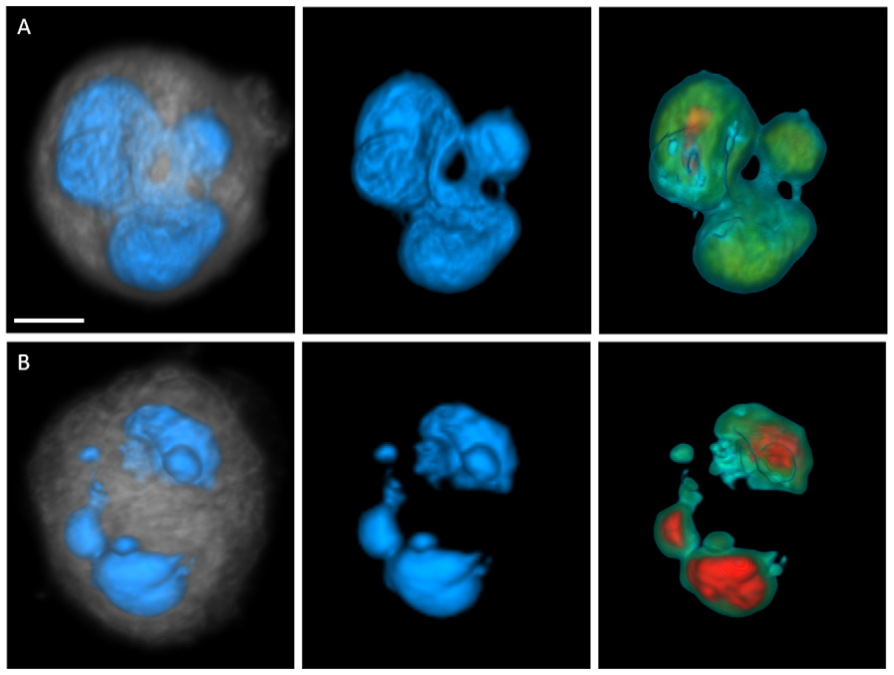
Instances of irregular MDA-MB-231 nuclear morphologies (scale bar = 5 microns). Panel (A) depicts a multilobular nucleus and panel (B) illustrates a cell with micronuclei. Left images show nuclear surface in blue and cytoplasm in gray, middle images show surface-shaded renderings of the nuclear volume, and right images depict volume renderings through the nuclear volume. Increasing nuclear density is color coded from green to red.

Figure 3A shows a representative instance of our occasional observation of distorted, lobulated nuclei in the MDA-MB-231 cells. Observations of similar nuclear shapes have alternately been described as polylobulated in the literature [32]. Figure 3B shows typical micronuclei, again an occasional phenomenon observed only in the MDA-MB-231 cell line. The images (and Movies S1, S2) reflected the uniquely powerful capability of micro-optical projection computed tomography to provide useful and effective visualization and precise quantification of 3D nuclear structure. 2D sectioning of multilobular nuclei or micronuclei could easily lead to one being mistaken for the other. It is almost impossible to reliably count nucleoli and quantify their margination from 2D imagery.

### 3D nuclear architecture varied from normal to metastatic but did not consistently correlate with existing nuclear grades

**Figure 4:**
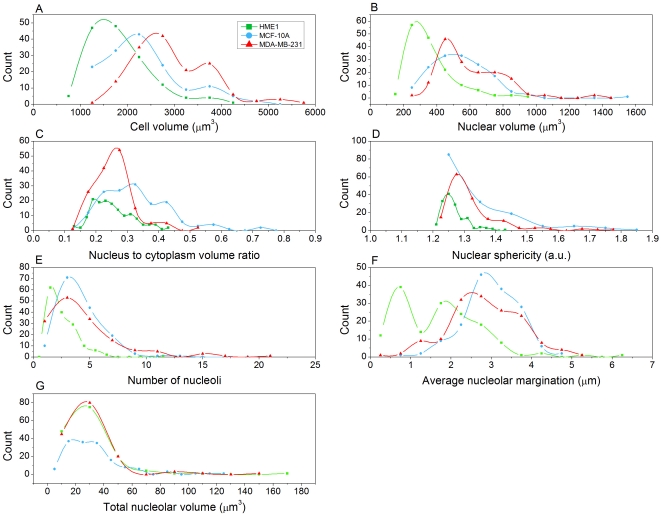
Histograms of morphological descriptors. Cubic splines (smooth curves) connect the dots (histogram bin centers).

We observed that the means of morphological parameters such as cell volume (Figure 4A) and nuclear volume (Figure 4B) increase in the order: HME1 (normal) < MCF-10A (fibrocystic disease) < MDA-MB-231 (metastatic adenocarcinoma). MCF-10A cells, however, had, on average, the largest nucleus-to-cytoplasm volume (N/C) ratio (Figure 4C). HME1 nuclei deviated the least from spherical shape (Figure 4D). The number (Figure 4E) and average margination of nucleoli – their tendency to be located proximal to the nuclear membrane - (Figure 4F) was higher in abnormal cells compared to the control. Most of the few nucleoli in normal cells were located close to the center of the nucleus. There was little difference in the total nucleolar volume between the three cell types (Figure 4G).

**Figure 5:**
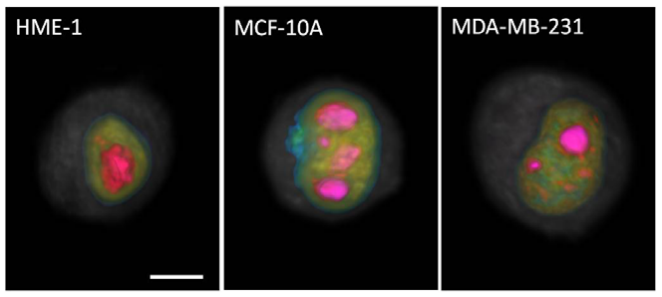
Variations in the organization of nuclear content between normal (left), fibrocystic (middle) and metastatic (right) mammary epithelial cells (scale bar = 5 microns). Light gray haze depicts cytoplasm. Nuclear membrane is blue, low density regions of chromatin are green, moderate density regions are yellow, high density regions are orange, and nucleoli are magenta. Increased clumpiness is apparent in abnormal cells, and metastatic cell contains large number of smaller high-density clumps. Opacity of nuclear membrane (in blue) has been deliberately decreased to enable viewing of nuclear interior. Corresponding videos of these renderings are available in Movies S3, S4, S5.

The variations in texture features among the cell lines captured noteworthy differences in patterns of coarse chromatin organization. Figure 5 shows a representative rendering of the distribution of different density regions within the nucleus to illustrate these textural variations (also see Movies S3, S4, S5). Figures S2, S3, S4 provide quantitative results relating to the texture features. MCF-10A and MDA-MB-231 cells had significantly higher total nuclear content than (Figure S2A) but similar mean voxel intensities (Figure S2B) to HME1 cells. The density variance among nuclear voxels in abnormal cells was lower than that for normal cells (Figure S2C). A chromatin clump is defined as a connected region, or contiguous cluster, of voxels falling within a density range. Abnormal cell nuclei contained a larger number of clumps (Figure S2D–F). The difference was more pronounced for the low and high density than for medium density clumps. Amorphous, connected medium-density regions often formed the surround for low- and high-density clumps (islands) of chromatin, especially in the abnormal cells. This was reflected in the histogram of Figure S2E. Most cells had one connected, medium density region. As shown in Figure S2G–I, abnormal cells had slightly lower volume fraction of low and high density regions compensated by a larger volume fraction of medium density regions. However, the differences in volume fractions of the three densities were much less pronounced than the differences in the numbers of clumps. The key point which can be deduced from the second and third rows of Figure S2 is that larger numbers of small low- and high-density clumps were present in abnormal compared to normal cells, which had smaller numbers of large clumps. The margination distributions of the different density clumps (Figure S3A–C) from the nuclear center of mass indicated that low density clumps were located peripherally in all cell types. High density clumps were predominantly close to the nucleus center in normal cell nuclei, whereas they were distributed somewhat more peripherally in abnormal cells. Comparison of Markovian texture feature distributions at the three selected granularity scales showed that MCF-10A nuclei exhibited the largest variations in density, MDA-MB-231 nuclei had density variations that are pronounced at finer scales, and HME1 nuclei also exhibited marked density variations (see Figure S4A–B). These results are in contrast to the popular notion that normal cells have a predominantly homogeneous nuclear interior. The variations in texture may be attributed to the transitions between the low-, medium-, and high- density regions in these nuclei. In contrast to the results from Figure S2D–F, Markovian feature distributions in Figure S4B suggest that density variations in nuclear content of metastatic cell nuclei are less prominent than in the normal cell nuclei. Both these inferences—that the nuclear interiors of normal cells are not homogeneous, and that metastatic cell nuclei are seemingly smoother than normal—can be accounted for by considering the small size of low- and high-density clumps in abnormal cell types. Definitive detection or classification based on texture may be confounded in abnormal cells exhibiting high Markovian similarity (see Figure S4B).

Statistical analyses enabled quantitative cross-validation of these descriptive assessments of the histogram distributions. The normality tests confirmed our visual assessment of the non-normality of a subset of feature data distributions (see Table S1). Table 2 shows the discriminatory power of the morphometric features. Using the ANOVA and Kruskal-Wallis tests and correcting for multiple comparisons, 90% (38/42) features were discriminatory between HME1 and both MCF10A and MDA-MB-231. This was reduced to 69% (29/42) of the features when discriminating between MCF10A and MDA-MB-231.

**Table 2:** Discriminatory power of features.

Feature name	Significance (p<0.0025, corrected for multiple comparison)	
	Normal v/s Abnormal	Fibrocystic v/s Metastatic
Cell volume	Yes	Yes
Nuclear volume	Yes	Yes
Nucleus to cytoplasm ratio	Yes	Yes
Nuclear sphericity	Yes	Yes
Number of nucleoli	Yes	No
Total nucleolar volume	No	No
Variance in nucleolar volume	Yes	No
Average nucleolar margination	Yes	No
Variance in nucleolar margination	Yes	Yes
Total nuclear content	Yes	Yes
Mean nuclear content	Yes	Yes
Variance in nuclear content	Yes	No
Skew in nuclear content	Yes	No
Kurtosis in nuclear content	Yes	Yes
Low density volume fraction	Yes	No
Medium density volume fraction	Yes	Yes
High density volume fraction	Yes	No
Low density content fraction	Yes	Yes
Medium density content fraction	Yes	Yes
High density content fraction	Yes	Yes
Number of low density clumps	Yes	Yes
Number of medium density clumps	Yes	Yes
Number of high density clumps	Yes	Yes
Low density compactness	Yes	Yes
Medium density compactness	Yes	No
High density compactness	Yes	Yes
Medium-high density compactness	Yes	Yes
Average extinction ratio (low-medium)	Yes	No
Average extinction ratio (low-high)	Yes	Yes
Average extinction ratio (low-mediumhigh)	Yes	Yes
Average distance from nucleus center to low density regions	Yes	Yes
Average distance from nucleus center to medium density regions	No	Yes
Average distance from nucleus center to high density regions	Yes	Yes
Average distance from nucleus center to medium-high density regions	Yes	Yes
Average centroidal distance from nucleus center to low density regions	Yes	Yes
Average centroidal distance from nucleus center to medium density regions	No	No
Average centroidal distance from nucleus center to high density regions	Yes	No
Average centroidal distance from nucleus center to medium-high density regions	No	No
Markovian contrast	Yes	Yes
Markovian correlation	Yes	Yes
Markovian energy	Yes	Yes
Markovian heterogeneity	Yes	Yes

## Discussion

Nuclear morphology and coarse chromatin organization are altered in cancer cells [32]–[34]. Computerized technologies that grade nuclei based on their morphometry have proven a powerful and objective tool to assess neoplastic progression [15], [35]–[38]. However, the anisotropic resolution inherent to traditional microscopy techniques precludes precise 3D structural characterization. Cell-CT provides the capability to visualize, study and accurately quantify 3D nuclear structure in individual cells.

We quantified variations in 3D nuclear structure in cell lines derived from three mammary epithelial cell types: normal, benign fibrocystic, and metastatic carcinoma. The use of standardized H&E staining protocols facilitates comparison of metrics computed in our study with conventional cytopathological assessments. The results of our analysis provide new perspectives on the morphological changes that may be associated with malignancy in breast cancer.

Our study is the first to report the predominant concave (mushroom cap) nuclear shape in mammary epithelial cells. The shape heterogeneity that we found in metastatic cell nuclei reflects the known large nuclear pleomorphism in invasive breast carcinoma [8]. The observed pleomorphism in normal cell nuclei is noteworthy and contradicts the conventional wisdom that normal cell nuclei exhibit little variability in shape. 2D sections through mushroom cap-shaped nuclei arrayed at random orientations on a slide would seriously under-represent the appearance of this distinctive shape, which may be a heretofore unrecognized characteristic of breast epithelial cell morphology. The marked shape distortions in metastatic cell nuclei appear to be indicative of increased ‘malleability’ as suggested by Zink et al. in [32] and may be linked to their metastatic capabilities. The shape distortions could also stem from the aneuploidy of the cell line, but more specific correlations are needed to establish this association.

The results of our morphometric analyses suggest intriguing conclusions. The increase in cell and nuclear volumes from normal to metastatic stage resulted in not-so-prominent differences in N/C ratio, a widely used metric in cancer detection. While the total nucleolar volume was similar in all studied cell types, descriptors such as the number of nucleoli and nucleolar margination toward the nuclear periphery showed marked variations between the normal and abnormal cells. Understanding the underlying reasons behind these trends may reveal insights into the functionality of nucleoli and its alteration in malignancy. While the increase in total nuclear content from normal to metastatic cells aligns with results from the literature about cancer cells having more DNA, the similarity in mean voxel intensities between the cell lines (Figure S2B) suggests that nuclear content increases proportionally with the nuclear size. The increased chromatin clumpiness in abnormal cells as illustrated in Figure 5, Figure S2D–F, and Movies S3, S4, S5 supports existing evidence, and may explain, in part, the observation by cytogeneticists that tumor chromatin has different properties that result in the characteristic difficulties in obtaining “good” G-banded chromosome preparations as compared to normal tissues. The observed variations in organization patterns of low-, medium-, and high-density regions between the cell types may correlate with transcription activity in these cells. Margination of high-density clumps in the abnormal and especially in metastatic cell nuclei suggests likely functional significance. It could indicate abnormally silenced chromatin. Probing protein expression in these regions using appropriate fluorescent markers may provide insights into epigenetic control mechanisms specifically activated in cancer cells.

The morphological similarity between fibrocystic and metastatic cells may not have been expected, since MCF10A cells have been used as controls in several research studies [39], [40]. Our statistical analysis revealed that diagnostically relevant descriptors such as number of nucleoli, nucleolar size or nucleolar margination toward the periphery may not be reliable parameters to distinguish malignant from fibrocystic cells. Larger Markovian texture variations in MCF-10A nuclei relative to the metastatic nuclei reflect a clumpier organization of nuclear content in the former, further confounding the ability of conventional morphological traits to distinguish these cell types. These findings point to new perspectives on the morphological changes associated with malignancy in breast cancer. It might be valuable to investigate in a future study whether induced over-expression of mutant p53 in the MCF-10A cells has any correlation with these observed morphological changes.

The variance observed in our morphometric feature distributions in Figure 4 and Figures S2, S3, S4 emphasizes the value of single-cell studies that permit quantitative assessment of intercellular heterogeneity. The cell-cell heterogeneity we observed could be attributed to differences in cell cycle phase. An interesting extension would be to repeat the studies using synchronized or sorted cell populations. Working with synchronized or sorted cells may also provide information about the effects of cell cycle on nuclear architecture and the concomitant morphological changes with malignancy. For data analysis, a useful next step would be to develop classifiers based upon the set of features with the most discriminatory power to distinguish among the different cell types, and to assess their generalizability to other tissues types and pathologies. The efficacy of commonly used classification techniques will require careful evaluation due to the non-normal feature data distributions.

A frequently cited drawback of cancer research based on immortalized cells from 2D culture is the absence of the extracellular matrix (ECM). In epithelial tumors, mutant parenchymal cells are embedded in and supported by connective tissue components and stromal cells. Bound and soluble proteins in the ECM, together with the connective fibrils, basement membrane and stromal cells, constitute the tumor microenvironment. Immortalized cancer cell line model systems may fail to recapitulate the natural signaling between ECM proteins and the nucleus. Communication between cancer cells and their microenvironment is now widely recognized to influence almost every important mechanism underlying cancer progression – including gene expression and tissue specificity – and several of the features relied upon for its diagnosis – including nuclear structure and chromatin architecture [41]. In spite of these drawbacks, cell line models of cancer progression provide easily accessible and readily reproducible means to develop tools, like high resolution 3D imaging, potentially useful for cancer diagnosis, and means to investigate drugs, potentially efficacious for its cure. We are pursuing additional research methodologies in efforts to circumvent some of the drawbacks of traditional immortalized cell culture model systems. One is growing cells in 3D culture [42]. A second is to disaggregate cells from animal or human biopsy specimens, grow them in primary cultures, and enrich for parenchymal cells before 3D imaging and analysis.

Genomic architecture drives nuclear structure and coarse chromatin organization. A wealth of information is now available about genomic alterations associated with neoplastic progression in breast cancer [43], [44]. Similar information is also available for a large number of breast epithelial cell lines [45]. However, relatively little is known about the genetic cues or mechanisms that govern nuclear organization patterns. A few nuclear matrix proteins (NMPs) have been shown to have different expression levels in breast cancer progression [46], and image-based investigations on the nuclear mitotic apparatus (NuMA) protein distribution in the nucleus using confocal microscopy are promising [47]. Accurate expression level quantification and 3D localization of the important nuclear proteins and their correlations with the measurements made in this study may reveal useful insights into nuclear organization and the mechanisms underlying its aberrations in cancer.

This information can be derived by 3D morphometric analysis of volumetric data generated from fluorescence-mode emission tomography. Fluorescence-mode cell-CT has the potential to provide precise spatial arrangement and expression level assessments for nuclear domains such as Cajal bodies, nucleoli, perinucleolar compartments, dense agglomerations of chromatin-organizing proteins, and PML bodies. By precisely registered complementary structural information obtained from absorption-mode 3D imagery, this data could contribute to more detailed descriptions of alterations in nuclear structure and understanding of the mechanisms causing them. We envision that our three-dimensional approach based on cell computed tomographic imaging will become a research and diagnostic tool capable of supplementing existing standard diagnostic procedures with quantitative, standardized spatial and functional data.

## Supporting Information

Figure S1 **Volume renderings of four shape categories (scale bar = 5 microns).**
(TIF)

Figure S2 **Histograms of textural descriptors.** Cubic splines (smooth curves) connect the dots (histogram bin centers). **a.u** refers to arbitrary units.
(TIF)

Figure S3 **Histograms of textural descriptors.** Cubic splines (smooth curves) connect the dots (histogram bin centers).
(TIF)

Figure S4 **Histograms of Markovian textural descriptors at three granularities.** Cubic splines (smooth curves) connect the dots (histogram bin centers).
(TIF)

Table S1 **Outcome of normality tests (Shapiro-Wilk test).**
(DOC)

Movie S1 **Multilobulated nucleus of metastatic carcinoma (MDA-MB-231) cell type.**
(WMV)

Movie S2 **MDA-MB-231 nucleus exhibiting micronuclei.**
(WMV)

Movie S3 **Optical density regions in normal mammary epithelial cell nucleus (scale bar = 5 microns).** Light gray haze depicts cytoplasm. Nuclear membrane is blue, low density regions of chromatin are green, moderate density regions are yellow, high density regions are orange, and nucleoli are magenta. Opacity of nuclear membrane (in blue) has been deliberately decreased to enable viewing of nuclear interior.
(WMV)

Movie S4 **Optical density regions in fibrocystic mammary epithelial cell nucleus (scale bar = 5 microns).** Light gray haze depicts cytoplasm. Nuclear membrane is blue, low density regions of chromatin are green, moderate density regions are yellow, high density regions are orange, and nucleoli are magenta. Opacity of nuclear membrane (in blue) has been deliberately decreased to enable viewing of nuclear interior.
(WMV)

Movie S5 **Optical density regions in metastatic mammary epithelial cell nucleus (scale bar = 5 microns).** Light gray haze depicts cytoplasm. Nuclear membrane is blue, low density regions of chromatin are green, moderate density regions are yellow, high density regions are orange, and nucleoli are magenta. Opacity of nuclear membrane (in blue) has been deliberately decreased to enable viewing of nuclear interior.
(WMV)
