# Supplementary figures and images for: Isotropic 3D Nuclear Morphometry of Normal, Fibrocystic and Malignant Breast Epithelial Cells Reveals New Structural Alterations
Source: PLoS One. 2012 Jan 5;7(1):e29230. doi: 10.1371/journal.pone.0029230 (PMC3252316; doi:10.1371/journal.pone.0029230)

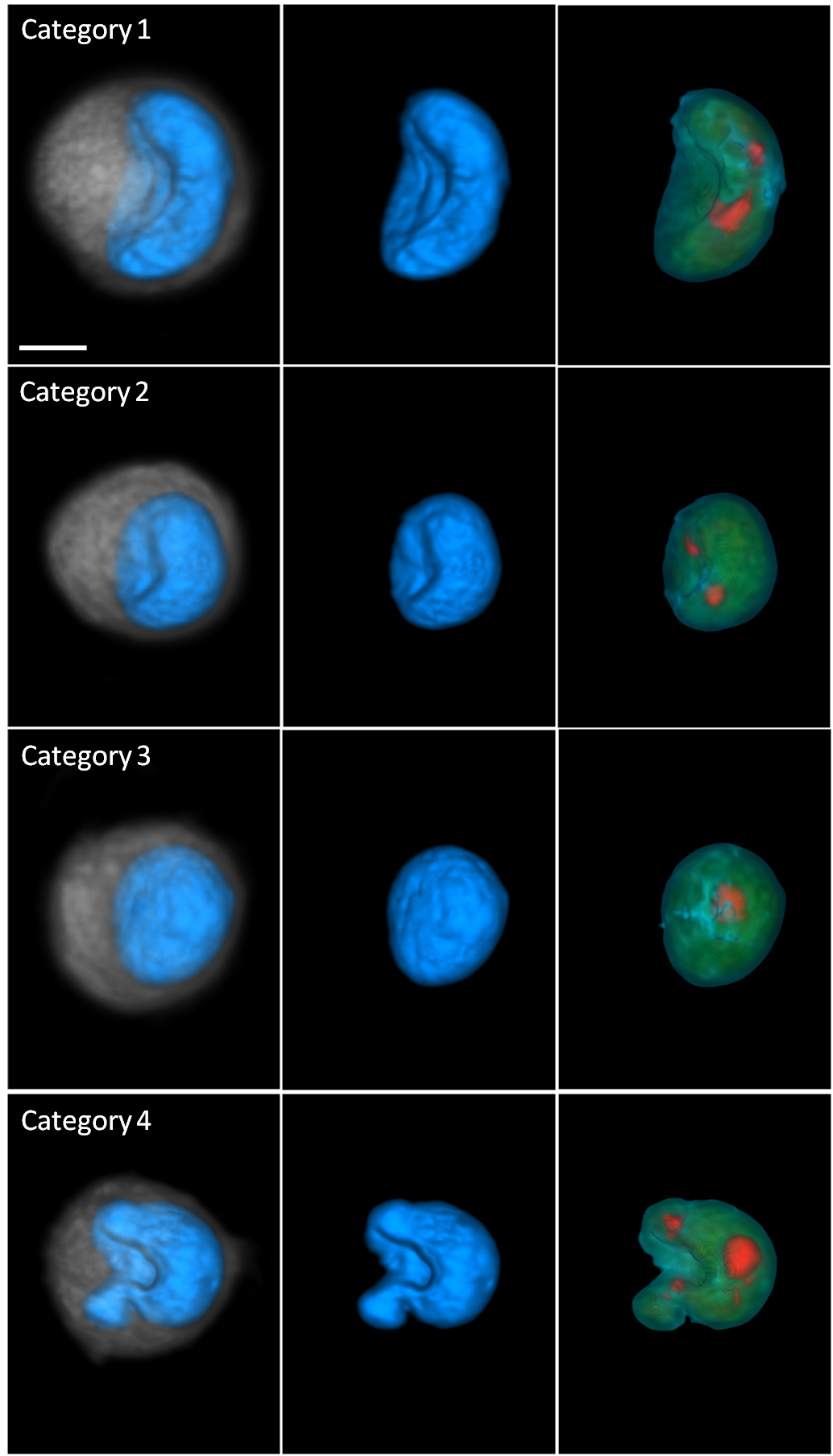

Supplement: Figure S1 — Volume renderings of four shape categories (scale bar = 5 microns). (TIF) [file pone.0029230.s001.tif]

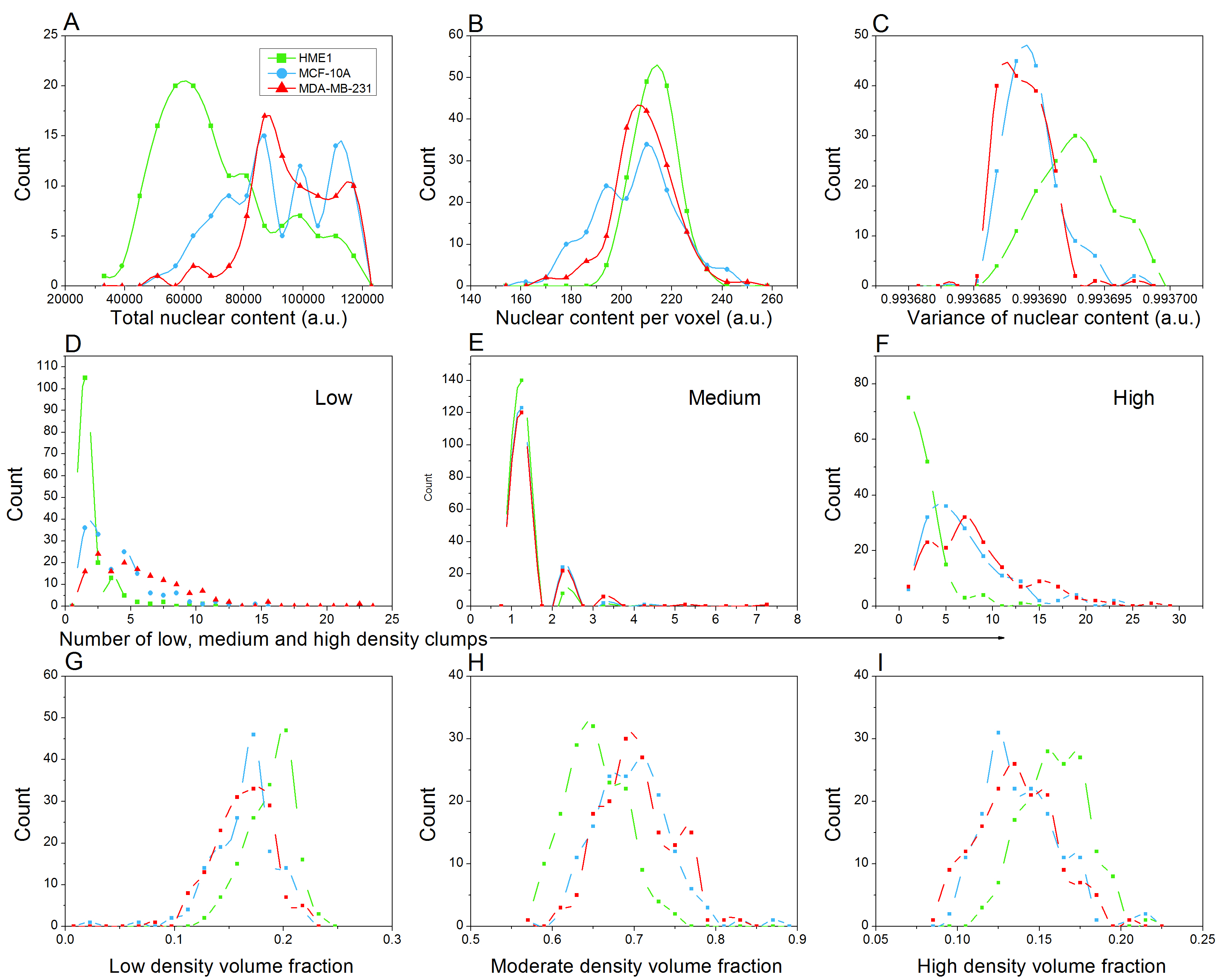

Supplement: Figure S2 — Histograms of textural descriptors. Cubic splines (smooth curves) connect the dots (histogram bin centers). a.u refers to arbitrary units. (TIF) [file pone.0029230.s002.tif]

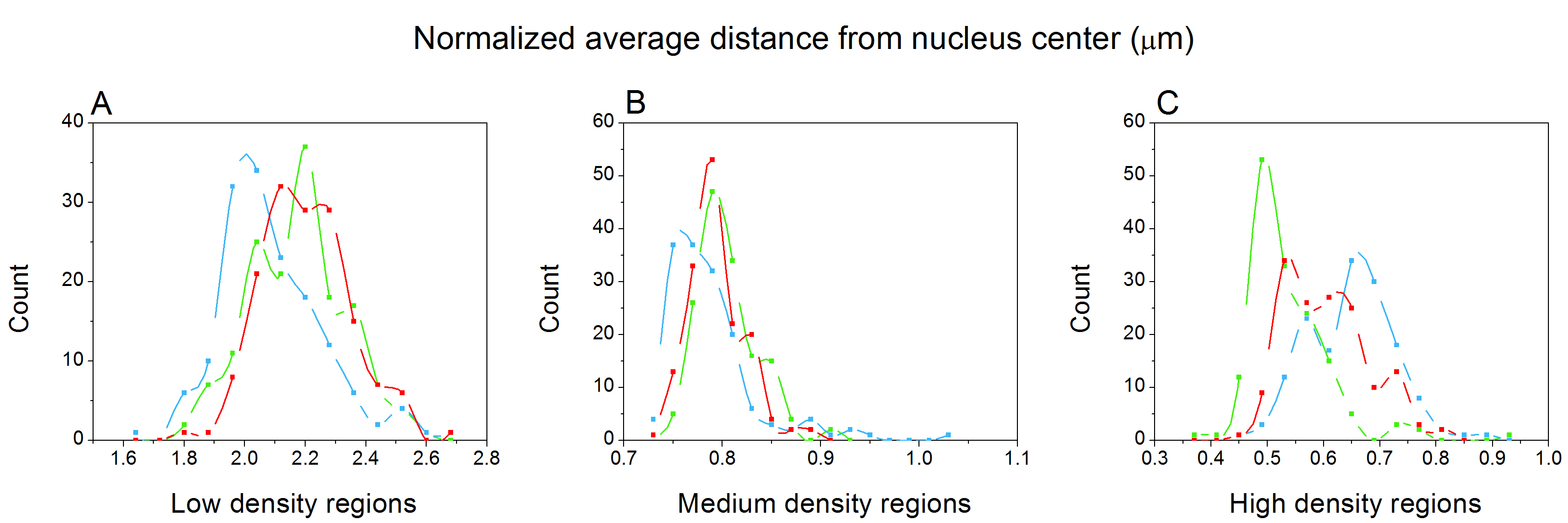

Supplement: Figure S3 — Histograms of textural descriptors. Cubic splines (smooth curves) connect the dots (histogram bin centers). (TIF) [file pone.0029230.s003.tif]

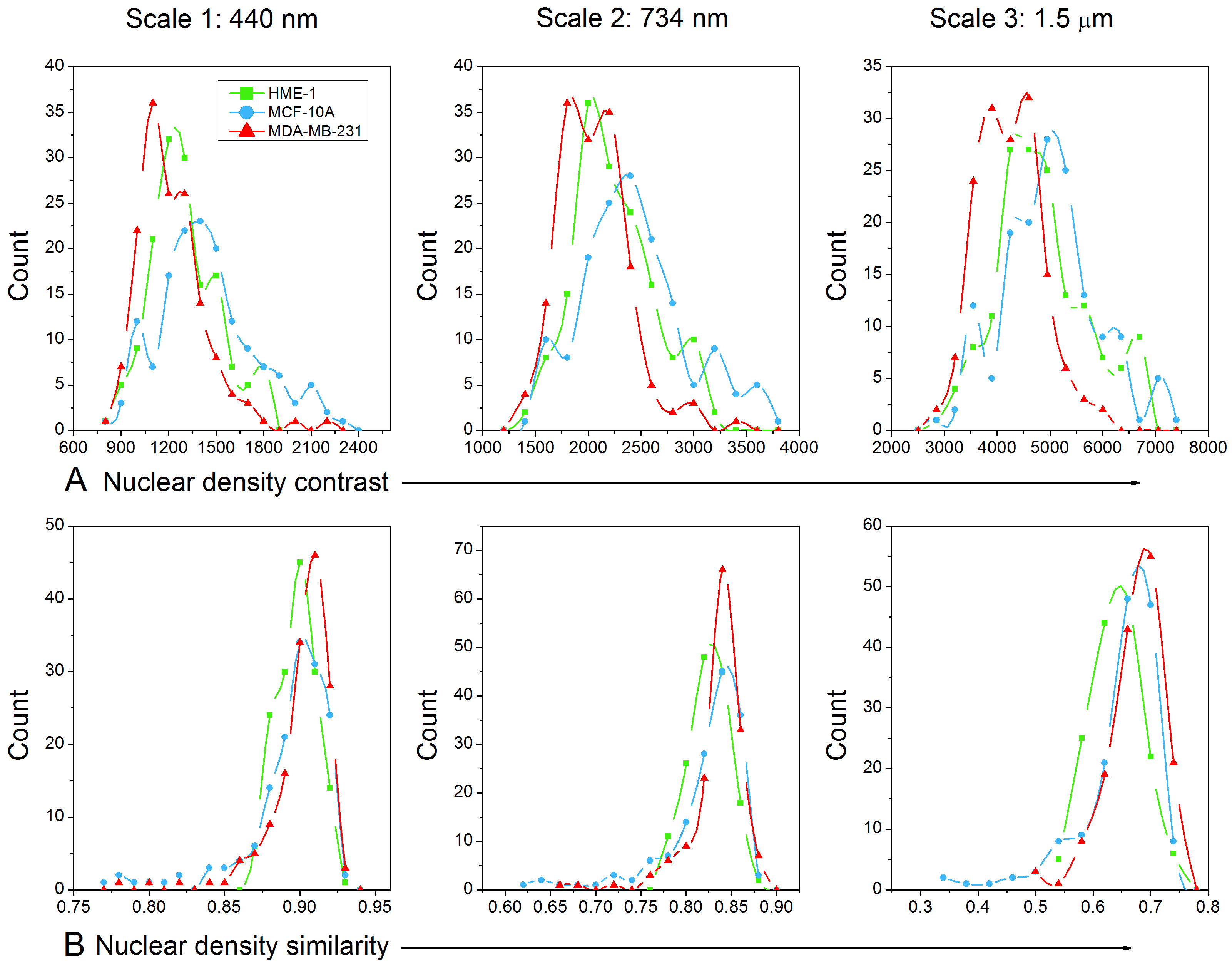

Supplement: Figure S4 — Histograms of Markovian textural descriptors at three granularities. Cubic splines (smooth curves) connect the dots (histogram bin centers). (TIF) [file pone.0029230.s004.tif]
