# Supplementary material for: Isotropic 3D Nuclear Morphometry of Normal, Fibrocystic and Malignant Breast Epithelial Cells Reveals New Structural Alterations
Source: PLoS One. 2012 Jan 5;7(1):e29230. doi: 10.1371/journal.pone.0029230 (PMC3252316; doi:10.1371/journal.pone.0029230)
Supplement: Table S1 — Outcome of normality tests (Shapiro-Wilk test). (DOC) [file pone.0029230.s005.doc]

**Supporting** Table 1: Outcome of normality tests (Shapiro-Wilk test)

| Features with normal distributions | Features with non-normal distributions |
| --- | --- |
|  |  |
| Mean nuclear content, medium density DNA fraction, medium density DNA content, Markovian energy (d = 3, 5, 10), Markovian homogeneity (d = 10) | Cell volume, nuclear volume, NC ratio, nuclear sphericity, number of nucleoli, total nucleolar volume, average nucleolar margination, total nuclear content, variance of nuclear content, high density DNA fraction, low density DNA fraction, low density DNA content, number of high density clumps, number of medium density clumps, number of low density clumps, high density compactness, medium density compactness, low density compactness, medium-high density compactness, average distance to from nucleus center to high, medium, low, medium-high density regions, average centroidal distance to high, medium, low, medium-low density regions, Average extinction ratios (low-high, low-medium, low-mediumhigh regions), Markovian contrast (d=3, 5, 10), Markovian correlation (d=3, 5, 10), Markovian homogeneity (d=3, 5) |
